# Supplementary material for: Chromothripsis during telomere crisis is independent of NHEJ, and consistent with a replicative origin
Source: Genome Res. 2019 May;29(5):737–49. doi: 10.1101/gr.240705.118 (PMC6499312; doi:10.1101/gr.240705.118)
Supplement: Supplemental Material [file supp_gr.240705.118_Supplemental_file_1.zip › contigs/annotated_contigs/DB55/contig.2.DB55_length_265_mean_cov_5.50188679245.docx]

**DB55_length_265_mean_cov_5.50188679245**

```
GGGAAGGCTGAGGCAGGAGAATCACTTGAACCTGGGAGGCGGAG|GG|AGCCTTAGGCAGCGGTAGGAGGTGACTTCCCACTCTGAGCA
 >chrUn_gl000220:121887-121933 - E=2e-16     >chr19:18909182-18909358 - E=5e-90
TCCAGTGACCAAATTGAGGAGACAGGACAGACTTGCTGAGCCTCAGTTTGCCAACCTGTAAAATGGGGATGTTAATAGCTGACATTGAT

TGAGGGCTCAGCTCTTTCTTTTCCTTTTTTTTTTTCTTTTTTT|GGTGCAGGGTTTTACTTGGGGTTACTCTGTGCGCCCAGGGTGGGG

T
```
